# The V sign in lateral talar process fractures: an experimental study using a foot and ankle model

**DOI:** 10.1186/s12891-017-1642-x

**Published:** 2017-07-03

**Authors:** Thorsten Jentzsch, Anita Hasler, Niklas Renner, Manuel Peterhans, Reto Sutter, Norman Espinosa, Stephan H. Wirth

**Affiliations:** 1 Department of Orthopaedics, Balgrist University Hospital, University of Zurich, Forchstrasse 340, 8008 Zurich, Switzerland; 2 Department of Orthopaedics, Kantonsspital Aarau, Aarau, Switzerland; 3 Department of Radiology, Balgrist University Hospital, University of Zurich, Zurich, Switzerland; 4 Institute for Foot and Ankle Reconstruction Zurich, Zurich, Switzerland

**Keywords:** Orthopedics and biomechanics, Biomechanics, Imaging, Ankle, Bone, Fracture

## Abstract

**Background:**

Lateral talar process fractures (LTPF) are often missed on conventional radiographs. A positive V sign is an interruption of the contour of the LTP. It has been suggested, but not proven to be pathognomonic for LTPF. The objective was to study whether the V sign is pathognomonic for LTPF and if it can be properly assessed in different ankle positions and varying fracture types.

**Methods:**

An experimental study was conducted. Two investigators assessed lateral radiographs (*n* = 108) of a foot and ankle model. The exposure variables were different ankle positions and fracture types. The primary outcome was the correct detection of a V sign. The secondary outcomes were the detection of the V sign depending on ankle position and fracture type as well as the uncertainty.

**Results:**

The interobserver agreement on the V sign and type of fracture were fair (κ = 0.35, 95% CI 0.18–0.53, *p* < 0.001 and κ = 0.37, 95% CI 0.26–0.48, *p* < 0.001). The mean sensitivity, specificity, PPV, NPV, and likelihood ratio for the detection of the V sign were 77% (95% CI 67–86%), 59% (95% CI 39–78%), 85% (95% CI 75–92%), 46% (95% CI 29–63%), and 2. The mean uncertainty in the V sign detection was 38%. The V sign identification stratified by ankle position and fracture type showed significant better results with increasing inversion (*p* = 0.035 and *p* = 0.011) and type B fractures (*p* = 0.001 and *p* = 0.013).

**Conclusions:**

The V sign may not be pathognomonic and is not recommended as the only modality for the detection of LTPF. It is better visualized with inversion, but does not depend on plantar flexion or internal rotation. It is also better seen in type B fractures. It is difficult to detect and investigator-dependent. It may be helpful in a clinical setting to point into a direction, but a CT scan may be used if in doubt about a LTPF.

## Background

Talar fractures constitute less than 1% of all fractures and less than 10% of fractures of the foot [1]. They are usually caused from high-energy trauma. Talar body fractures (61%) are more common than neck and head fractures [2]. Among all body fractures, dome compression and lateral process fractures (24%) are the most common and missed types when using conventional radiography. Lateral talar process fractures (LTPF) are rare, but account for 15% of ankle injuries in snowboarders, who reveal a 17-fold increased risk of sustaining such a fracture [3, 4]. They are caused by forced dorsiflexion, inversion, and potentially external rotation and are commonly missed on anteroposterior and lateral radiographs [5, 6]. In 1965, Hawkins introduced his classification system of these fractures [7]. Type I (42%) fractures are more common than type II (32%) and type III (24%) fractures [8]. Type I and II fractures involve the talofibular and posterior subtalar joint, while type III fractures involve the posterior subtalar joint only. Type I is defined as a simple fracture. Type II constitutes a comminuted fracture of the entire process. Type III is an anterior-inferior chip fracture. Type I fractures can be further subdivided according to displacement, with <2 mm in type IA and ≥2 mm in type IIB types. It is vital to diagnose these fractures early to avoid long-term morbidity due to painful arthritis and avascular necrosis.

Usually, the radiographic workup consists of anteroposterior, dorsoplantar, mortise, and lateral views of the foot and ankle. Lateral views of normal ankles demonstrate a V-shaped lateral talar process. Displaced fractures show an interrupted V shape [9]. In other words, radiographic appearance of the lateral talar process resembles an uninterrupted (symmetric) V-shape in healthy individuals, but it appears as an interrupted (asymmetric) V-shape in patients with LTPF. Therefore, it has been suggested, but not proven, that a positive V sign is pathognomonic for displaced LTPF. In theory, the V sign could be important when assessing potential fractures that may occur after ankle sprains [10].

However, the literature on the V sign is sparse and lacks a systematic approach [9, 10]. In clinical emergency practice, standardized weight bearing lateral radiographs are often insufficient for proper ankle assessment. Due to pain and functional deficits, including the inability to bear weight, as well as accompanying ankle injuries and variable imaging techniques, lateral radiographs are highly susceptible to rotational malpositioning. Hypothetically, this could negatively affect the presence of the V sign in normal ankles. In addition, the severity of fractures may influence the prevalence of the V sign as well.

The aim of the study was to investigate the performance of V sign assessment under different ankle positions and varying fracture patterns of the lateral talar process. The null hypothesis was that the V sign is always present in LTPF and that it keeps unchanged when assessed at different positions of the ankle and varying fracture types.

## Methods

### Study design and setting

An experimental study was conducted at a University Hospital in Switzerland in February 2016 to compare the detection of the V sign in normal and fractured lateral talar processes according to different ankle joint positions and fracture types. A waiver was granted from the local ethics committee (Kantonale Ethikkommission Zürich, BASEC-Nr. Req-2017-00429) and written informed consent was obtained from the patient that is presented as a clinical example.

### Model and investigators

**Fig. 1:**
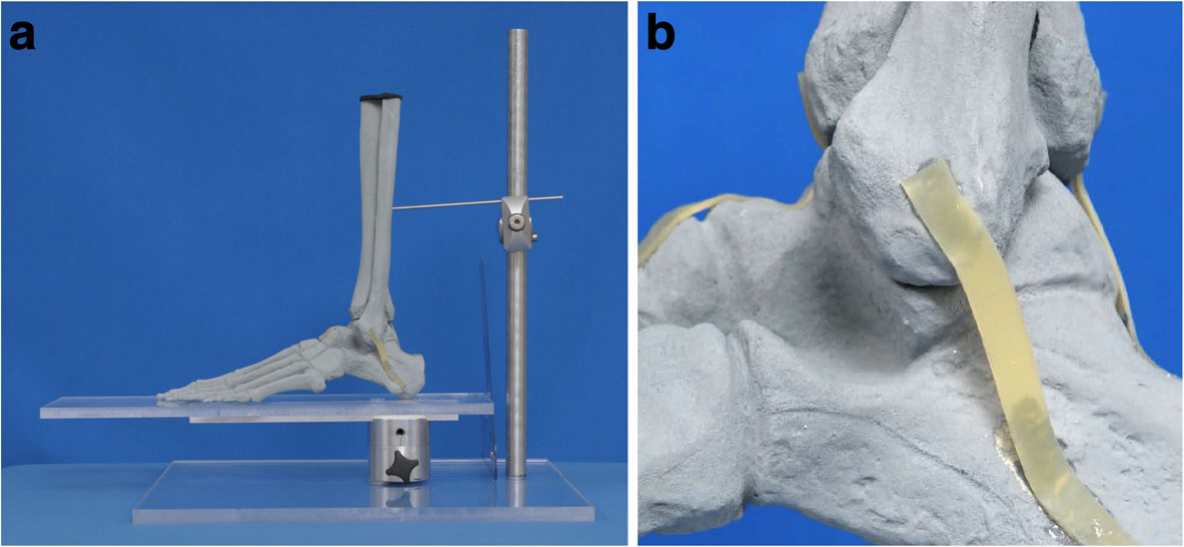
Photographs of study set up with a mounted distal foot and ankle sawbone model showing an intact lateral talar process. **a** Overview. **b** Hindfoot including lateral talar process

**Fig. 2:**
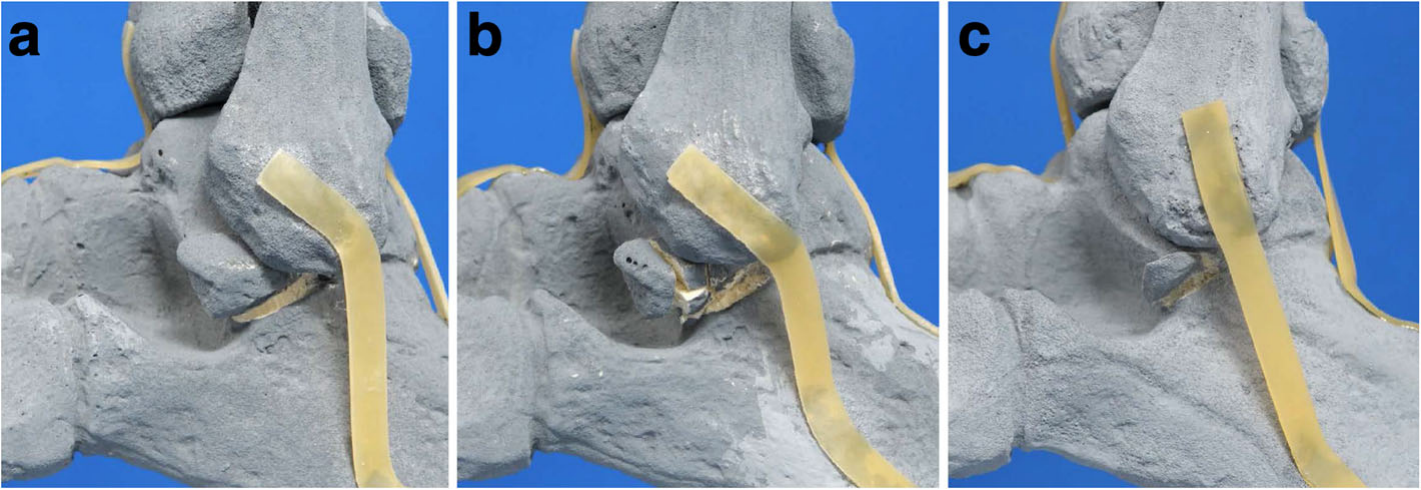
Photographs of lateral talar process fractures of the distal foot and ankle model. **a** Type A fracture. **b** Type B fracture. **c** Type C fracture

Four left, large, high resolution, solid foam, radiopaque distal foot and ankle sawbones (Pacific Research Laboratories, Vashon, WA, USA) were used in this model (Fig. 1). These were evaluated by a fellow and consultant in orthopedic surgery. The gold standard was defined by both investigators, who obtained the x-rays and knew the macroscopic appearance of the model. It reflected the true nature of the model, where the V sign was absent in all intact lateral talar processes (Fig. 1) and present in all LTPF (Fig. 2).

### Variables

Exposure variables included varying ankle joint positions during lateral radiographs and different fracture types. First, varying degrees of plantar flexion (0°, 20° and 40°) were examined. Second, varying degrees of inversion (0°, 20° and 40°) were added [11]. Third, varying degrees of internal rotation (0°, 20° and 40°) were added [6]. These angles were chosen according to physiological values for the range of motion of the ankle joint, common relieving postures during ankle pain, and previous reports on mechanisms of injury [6, 11]. There were four different fracture models: no fracture, type A, type B, and type C. This resulted in 27 different radiographs for each group.

The primary outcome variable was the correct detection of a V sign [9]. The secondary outcome variables were the detection of the V sign depending on the ankle position and fracture type according to Hawkins as well as the uncertainty in the evaluation of the V sign [7].

### Data sources and measurements

**Fig. 3:**
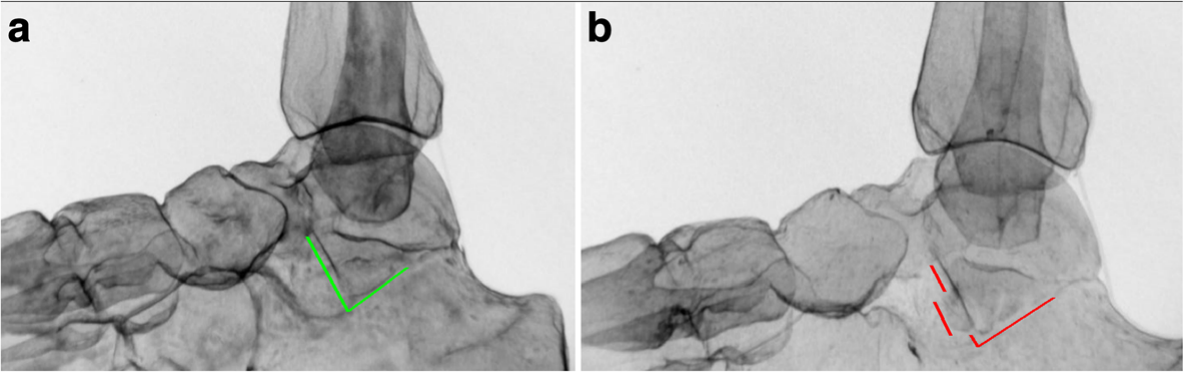
Illustration of the V sign. **a** Negative (absent) V sign, which is illustrated by the intact (symmetric) V-shape of the lateral talar process depicted in *green*. **b** Positive (present) V sign, which is illustrated by the defective (asymmetric) V-shape of the lateral talar process depicted in *red*

**Fig. 4:**
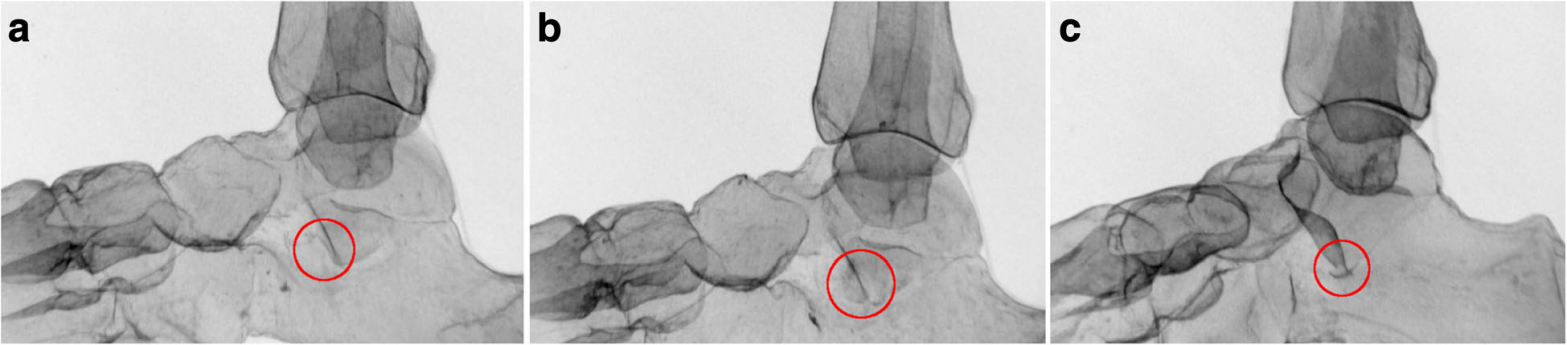
The V sign on lateral radiographs, as demonstrated in the sawbone models. The *circles* indicate the location of the V sign. **a** Positive V sign; type A fracture. **b** Positive V sign; type B fracture. **c** Positive V sign; type C fracture

One model was left intact, while the other three models had displaced (5 mm) fractures introduced with increasing severity to resemble different fracture patterns according to the Hawkins classification [7] (Fig. 2). A chisel and hammer were used to create these fractures and double-faced adhesive tape (Beiersdorf AG, Tesa SE, Norderstedt, Germany) was needed to hold these fractures in place. The V sign is illustrated in Fig. 3. Lateral radiographs were acquired for each model and each position by two investigators (Fig. 4). Two other investigators documented their decisions about the V sign in REDCap (version 6.11.5; Vanderbilt University, Nashville, TN, USA).

### Radiographic technique

Lateral radiographs were acquired with a fully digital radiographic C-arm system (Ziehm Vision; Ziehm Imaging GmbH, Nurnberg, Germany). The tube voltage was 44 kV, the tube load 1.9 mAs, and the acquisition duration 2 s. Models were placed as close as possible to the image intensifier.

### Bias

Choosing different independent investigators for the acquisition and review of radiographs as well as blinding of the latter minimized observer bias. Information bias was addressed by randomizing and numbering the radiographs from 1 to 108 using random number tables (Excel, version 2010; Microsoft Corporation, Redmond, USA). Errors in classifying the radiographs were minimized by training the investigators how to assess the V sign according to the technique published in previous reports [9, 12]. Both investigators received the same instruction prior to the beginning of the study. For this purpose, the investigators received detailed information about the V sign. In addition, examples of radiographs with negative and positive V signs using clinical and model radiographs helped to improve the training sessions.

### Calculation of study size

A total of 108 lateral radiographs were obtained. It was assumed that the V sign can be seen in the majority (90%) of radiographs from the fracture group (*n* = 81), but remains mostly absent (10%) in radiographs of the normal group (*n* = 27). This would require a minimum of five samples per group and a total number of 10 samples for a power of 80% and a significance level of 5%. The chosen study size surpassed these calculations.

### Statistical methods

Data are presented as absolute numbers and their percentages. Groups were compared with the chi-squared test for categorical values. The interobserver measure of agreement was calculated with the kappa (κ) coefficient. Ninety-five percent confidence intervals were calculated with the formula ‘estimate ± 1.96 × standard error’. The sensitivity, specificity, negative predictive value (NPV), positive predictive value (PPV), and likelihood ratio are presented for the V sign. Exact 95% confidence intervals are presented using means of numerators and denominators for both investigators, respectively. New binary variables were created for the correct detection of a V sign for each investigator. There were no missing values. The significance level was set at 5%. Statistical analysis was performed with Stata/IC (version 13.1; StataCorp LP, College Station, TX, USA).

## Results

### Participants

The interobserver agreement on the V sign and type of fracture were fair (κ = 0.35, 95% CI 0.18–0.53, *p* < 0.001 and κ = 0.37, 95% CI 0.26–0.48, *p* < 0.001, respectively) (Tables 1 and 2). The mean uncertainty of both investigators about the presence of the V sign was 38%. The agreement on the uncertainty of the presence of the V sign and type of fracture were moderate and fair, respectively (κ = 0.51, 95% CI 0.32–0.70, *p* < 0.001 and κ = 0.36, 95% CI 0.18–0.54, *p* < 0.001, respectively).

**Table 1:** Detection of the V sign by each investigator

V Sign Detection: Investigator 1	V Sign Detection: Investigator 2			Kappa* (95% CI)	*P*-value^†^
	No (n [%])	Yes (n [%])	Total (n [%])		
No	19 (70)	23 (28)	42 (39)	0.35 (0.35–0.42)	<0.001
Yes	8 (30)	58 (72)	66 (61)		
Total	27 (100)	81 (100)	108 (100)		

*Interobserver measure of agreement

^†^Kappa statistic

**Table 2:** Detection of the fracture type by each investigator

Fracture Type Detection: Investigator 1	Fracture Type Detection: Investigator 2					Kappa* (95% CI)	*P*-value^†^
	No fracture (n [%])	Type A (n [%])	Type B (n [%])	Type C (n [%])	Total (n [%])		
No fracture	26 (60)	9 (38)	3 (13)	4 (22)	42 (39)	0.37 (0.24–0.50)	<0.001
Type A	7 (16)	12 (50)	6 (26)	2 (11)	27 (25)		
Type B	6 (14)	3 (13)	11 (48)	2 (11)	22 (20)		
Type C	4 (9)	0 (0)	3 (13)	10 (56)	17 (16)		
Total	43 (100)	24 (100)	23 (100)	18 (100)	108 (100)		

*Interobserver measure of agreement

^†^Kappa statistic

The mean sensitivity, specificity, PPV, and NPV for the detection of the V sign were 77% (95% CI 67–86%), 59% (95% CI 39–78%), 85% (95% CI 75–92%), and 46% (95% CI 29–63%) (Table 3). The prevalence of a V sign was 75% (81 V signs [and fractures] and 27 absent V signs [no fractures]). The pretest odds, likelihood ratio, and posttest odds were 3, 2, and 6, respectively.

**Table 3:** Performance of the V sign

	Result (% [95% CI*])
Sensitivity	77 (67–86)
Specificity	59 (39–78)
NPV	85 (75–92)
PPV	46 (29–63)

*Exact 95% confidence intervals are presented using means of numerators and denominators for both investigators, respectively

The correct identification of the V sign stratified by ankle position showed significant better results with increasing inversion (*p* = 0.035 and *p* = 0.011 for each investigator, respectively) (Table 4). It was not associated with plantar flexion and internal rotation (*p* = 0.31 and 0.33 for plantar flexion as well as 0.35 and 0.53 for internal rotation for each investigator, respectively). With an inversion of 40°, the V sign was correctly identified in 83 and 89%; without inversion, in 56 and 61%. Furthermore, the correct identification of the V sign stratified by fracture type revealed significant differences between fracture types (*p* = 0.001 and *p* = 0.013 for each investigator, respectively) (Table 5). The V sign was correctly identified most often in fractures types B (89% each), while the V sign was detected least often in cases without a fracture (63 and 56%, for each investigator, respectively).

**Table 4:** Correctly identified V sign stratified by investigator and ankle position

Correctly Identified V Sign	Ankle position (°)			Total	*P*-value^*^
	0 (n [%])	20 (n [%])	40 (n [%])		
	Inversion				
Investigator 1					
No	16 (44)	13 (36)	6 (17)	35 (32)	0.035
Yes	20 (56)	23 (64)	30 (83)	73 (68)	
Total	36 (100)	36 (100)	36 (100)	108 (100)	
Investigator 2					
No	14 (39)	6 (17)	4 (11)	24 (22)	0.011
Yes	22 (61)	30 (83)	32 (89)	84 (78)	
Total	36 (100)	36 (100)	36 (100)	108 (100)	
	Plantar flexion				
Investigator 1					
No	15 (42)	11 (31)	9 (25)	35 (32)	0.31
Yes	21 (58)	25 (69)	27 (75)	73 (68)	
Total	36 (100)	36 (100)	36 (100)	108 (100)	
Investigator 2					
No	10 (28)	9 (25)	5 (14)	24 (22)	0.33
Yes	26 (72)	27 (75)	31 (86)	84 (78)	
Total	36 (100)	36 (100)	36 (100)	108 (100)	
	Internal rotation				
Investigator 1					
No	10 (28)	15 (42)	10 (28)	35 (32)	0.35
Yes	26 (72)	21 (58)	26 (72)	73 (68)	
Total	36 (100)	36 (100)	36 (100)	108 (100)	
Investigator 2					
No	6 (17)	10 (28)	8 (22)	24 (22)	0.53
Yes	30 (83)	26 (72)	28 (78)	84 (78)	
Total	36 (100)	36 (100)	36 (100)	108 (100)	

^*^Chi-squared test

**Table 5:** Correctly identified V sign stratified by investigator and fracture type

Correctly Identified V Sign	Fracture Type					*P*-value^*^
	No Fracture (n [%])	Type A (n [%])	Type B (n [%])	Type C (n [%])	Total (n [%])	
Investigator 1						
No	10 (37)	16 (59)	3 (11)	6 (22)	35 (32)	0.001
Yes	17 (63)	11 (41)	24 (89)	21 (78)	73 (68)	
Total	27 (100)	27 (100)	27 (100)	27 (100)	108 (100)	
Investigator 2						
No	12 (44)	4 (15)	3 (11)	5 (19)	24 (22)	0.013
Yes	15 (56)	23 (85)	24 (89)	22 (81)	84 (78)	
Total	27 (100)	27 (100)	27 (100)	27 (100)	108 (100)	

^*^Chi-squared test

## Discussion

On conventional lateral radiographs, the lateral talar process has an uninterrupted (symmetric) V-shape in healthy individuals, but an interrupted (asymmetric) V-shape in patients with LTPF. In the present study, the interobserver agreement on the V sign and type of a fracture were only fair, demonstrating that the V sign and sole use of lateral radiographs are of limited use in the clinical setting. With a mean uncertainty about the presence of the V sign of 38%, the V sign was difficult to evaluate. While the sensitivity of 77% for the detection of the V sign may be considered somewhat useful, the specificity was too low. A likelihood ratio of 2 indicated that the V sign increased the probability of a LTPF by approximately 15% [13]. The V sign is also more likely to be detected if the ankle assumes an inverted position and in cases of type B fractures, while plantar flexion and internal rotation do not seem to influence its detection. These findings are based on conventional lateral radiographs and no specific radiological view is necessary for better demonstration. If in doubt about the presence or absence of a LTPF, a CT scan may be useful.

In the literature, there are two studies about the V sign. First, in a retrospective cohort study of 23 snowboarders with a mean follow-up of 3.5 years after conservative and surgical treatment, the American Orthopedic Foot and Ankle Society (AOFAS) hindfoot score was 94 (of 100) points, but subtalar osteoarthritis was found in 45% [9]. It was concluded that the outcome was favorable if the diagnosis was established early and the treatment was adequately tailored to the displacement as well as associated injuries. To establish early diagnosis, a positive pathognomonic V sign was mentioned. Second, a case report described a 31-year old women with a LTPF, originally misdiagnosed as an ankle sprain despite the pathognomonic presence of a V sign [10]. Before using these findings in a clinical setting with confidence, further studies evaluating in vivo findings may be needed.

Conventional radiographic suspicion of LTPF is usually confirmed with computed tomography (CT) to evaluate the extent of the fracture. If CT scans are not available or during intraoperative assessment, the Canale and Harris views may be added to visualize the talar neck and subtalar joint. The mortise view (internal rotation of the ankle by 20°) prevents overlapping of the talus with the fibula for assessment of the lateral talus. Treatment of LTPF usually consists of conservative measures for nondisplaced type III fractures, excision for displaced (>2 mm) fractures with multiple small fragments (type II), and internal fixation for displaced fractures with a large (≥1 cm) fragment (type I) [8, 11, 14, 15].

Selection bias may have been introduced by aberrant fracture patterns resulting in non-differential misclassification of (non-)fracture types potentially under- or overestimating the strength of association. Furthermore, the use of a foot and ankle model may not accurately reflect the setting in humans. However, it is a feasible method for standardized evaluation of the ankle joint without causing radiation and potential pain to patients with ankle injuries.

**Fig. 5:**
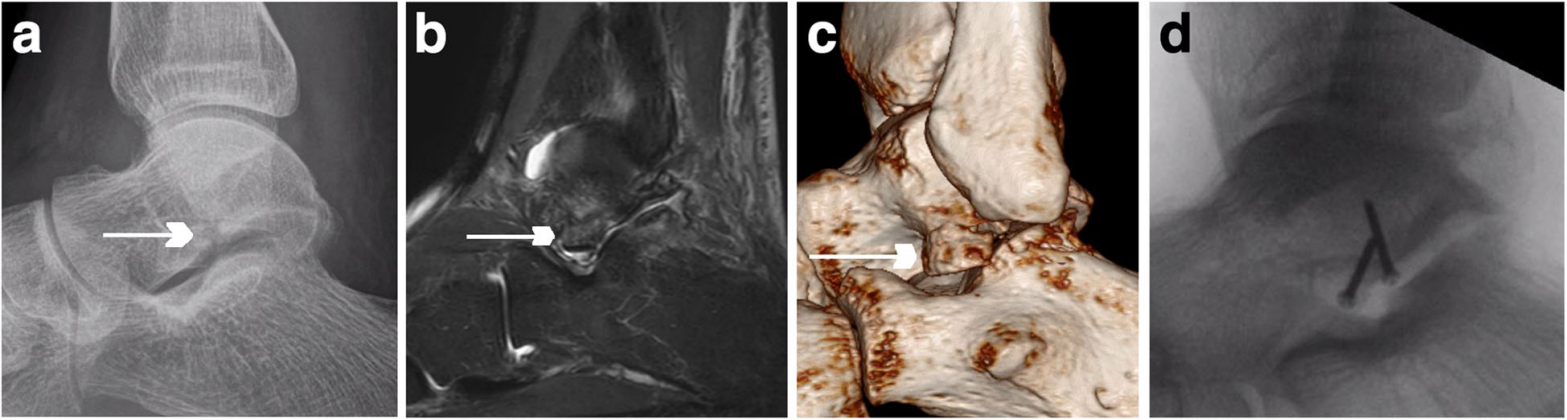
Clinical example of a patient with a lateral talar process fracture (LTPF). **a** Conventional lateral radiograph. **b** MRI showing a sagittal proton density fat-saturated image, which demonstrates the fracture line (*arrow*). **c** Computed tomography images confirming the displaced lateral talar process fracture (*arrows*). **d** Conventional lateral radiograph after open reduction and internal fixation with two screws

As a clinical example, we briefly report on a 41 year-old male patient with a LTPF after a snowboard accident 1 month ago (Fig. 5). Conventional radiographs were initially obtained at an external institution before he was treated for a low ankle sprain. Due to persisting pain after 1 month, an MRI was obtained and he was referred to our institution for further evaluation. This demonstrated the type I LTPF and adjacent bone marrow edema. Radiographs and computed tomography images were acquired for better assessment of the osseous structures and to confirm the displaced lateral talar process fracture. The fracture was treated with an open reduction and internal fixation with two screws followed by non-weightbearing in a cast for 6 weeks.

In daily practice of every physician that treats traumatic foot and ankle injuries, the first step in the diagnostic cascade after clinical evaluation usually includes the acquisition of a conventional radiograph. The results of this novel experimental study demonstrate that standard lateral x-rays of the foot and ankle are somewhat helpful to avoid missing a commonly missed LTPF and to strengthen the suspicion of a LTPF. If tolerated by patients, the visualization of LTPF may be enhanced by inverting the foot without the need to change flexion or rotation. These findings are particularly interesting to physicians and regions where CT scans or MRIs are not readily available. If the V sign is positive or there is negative V sign but high clinical suspicion of a LTPF, patients may benefit from CT scans or MRIs for better evaluation of the type of fracture and size of fragments in order to plan the best treatment strategy for each patient.

## Conclusions

We conclude that the V sign may not be pathognomonic and is not recommended as the only modality for the detection of a LTPF. It is better visualized with inversion of the foot, but does not depend on plantar flexion or internal rotation. It is also better seen in type B fractures. It is difficult to detect and investigator-dependent. It may be helpful in a clinical setting to point into a direction, but a CT scan may be used if in doubt about a LTPF.

## Abbreviations

LTPF: Lateral talar process fractures
